# Vulva Fibroadenoma Associated with Lactating Adenoma in a 26-Year-Old Nigerian Female

**DOI:** 10.1155/2013/195703

**Published:** 2013-08-26

**Authors:** C. C. Anunobi, F. J. N. Obiajulu, A. A. F. Banjo, A. O. E. Okonkwo

**Affiliations:** ^1^Department of Anatomic and Molecular Pathology, Lagos University Teaching Hospital, PMB 12003, Lagos 101014, Nigeria; ^2^Angel Hospital, Lagos, Nigeria

## Abstract

*Background*. Vulva lactating adenoma is rare and may be misdiagnosed as adenocarcinoma in frozen section and aspiration cytology if breast tissue is not anticipated. *Objective*. To raise the awareness of lactating vulva ectopic breast lesion among clinicians and pathologists. *Case Report*. We present a case of vulva fibroadenoma associated with lactating adenoma in a 26-year-old Nigerian female. *Conclusion*. The rarity of vulva ectopic breast tissue can present a diagnostic challenge for both the clinician and the anatomical pathologist. Once excisional biopsy is done and the lesion confirmed histologically, the anxious patient can be reassured.

## 1. Introduction

Ectopic breast tissue is found along the primitive embryonic milk lines which extend from the axilla to the medial side of the groin [1]. Normally in humans, only a small part of the milk line persists in the mid-thoracic region. Incomplete involution anywhere outside this midthoracic region and along the primitive milk streak can result in ectopic mammary tissue. The reported incidence of ectopic breast in women is 2–6%, but only a few of such case reports arising from the vulva exist in the literature [2]. Deaver reported that Hartung in 1872 described the first fully formed mammary gland in the vulva [3]. Ectopic breast tissue responds to hormones and may undergo benign or malignant process as those of normally located breast tissue [4]. Although lactational changes can occur in ectopic breast, lactating adenoma in the vulva is extremely rare [4]. 

We describe a case of ectopic vulva breast tissue presenting as fibroadenoma associated with lactating adenoma in a breast feeding woman.

## 2. Case Report

A 26-year-old G_4_P_3_^+1^ woman presented to a peripheral hospital with a painless vulva swelling of 6 months duration in the second trimester of her pregnancy. The swelling was first noticed as a small nodule that grew appreciably in size during pregnancy and postpartum. Physical examination revealed a 4 cm × 2 cm mass located at the right lip of the labium majus. She delivered a set of twins after which the mass was excised and sent to the Department of Anatomic and Molecular Pathology of Lagos, University Teaching Hospital, Idi-Araba, Lagos for histological analysis.

**Figure 1:**
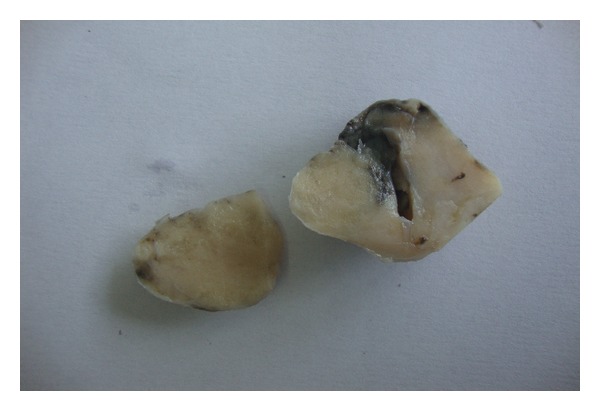
Encapsulated oval tissue with lobulated grayish white cut surface.

On gross examination, the specimen was an encapsulated piece of grayish white tissue measuring 3 cm × 2.5 cm × 2 cm. Cut section showed bulging lobulated grayish white surface (Figure 1).

The specimen was processed and evaluated with haematoxylin and eosin staining. 

**Figure 2:**
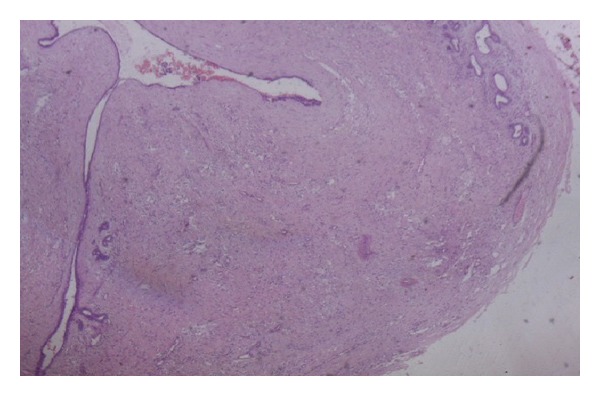
(H&E ×100) Encapsulated growth (fibroadenoma) consisting of compressed glands and ducts within a dense fibrocollagenous stroma.

**Figure 3:**
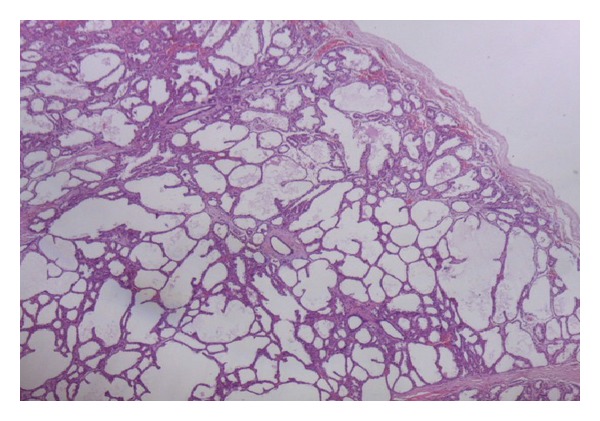
(×100) Encapsulated breast lesion showing dilated glands and ducts with secretory activity.

Histological sections showed an encapsulated breast tissue consisting of combined proliferation of epithelial and mesenchymal elements. The fibrocellular stroma proliferates around the tubular ducts. The ducts are of different sizes and shapes and are lined by inner columnar epithelial cells and outer myoepithelial cells. An area of lactating adenoma is seen with glands showing secretory activity. Large alveolar spaces separated by fine fibrovascular trabeculae are seen (Figures 2 and 3).

**Figure 4:**
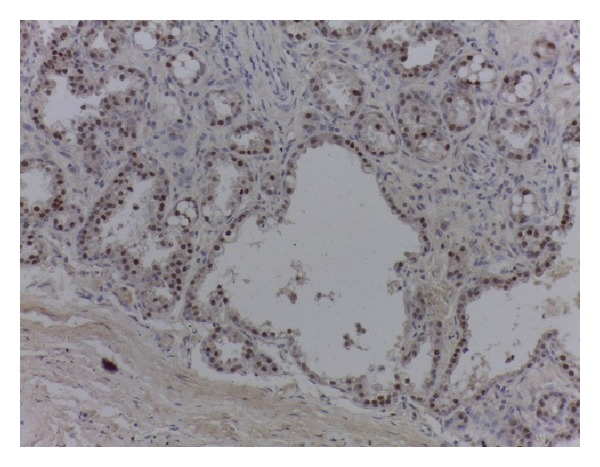
(×400) Ductal cells with strong estrogen receptor positivity.

**Figure 5:**
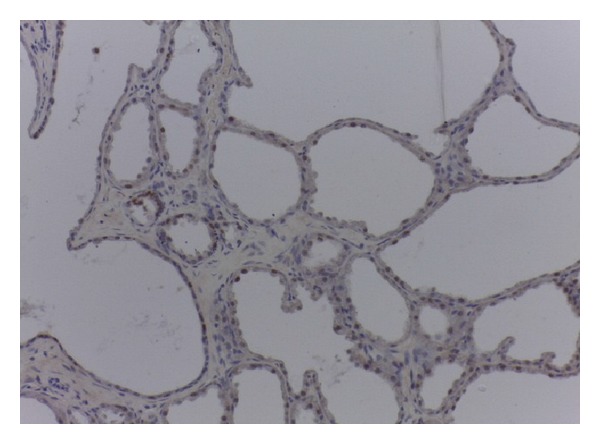
(×400) Ductal cells with weak progesterone receptor positivity.

Immunohistochemical studies were also done and the cells reacted positively for ER and PR (Figures 4 and 5). Avidin biotin complex type method with antibody dilution of 1 : 100 was used. 

A diagnosis of fibroadenoma associated with lactating adenoma occurring in a vulva ectopic breast tissue was made. The postoperative period was uneventful.

## 3. Discussion

Ectopic breast tissue in the vulva is a rare lesion [5]. This might be related to the fact that the vulva is located at the inferior end of the primitive mammary ridge. The lesions may undergo benign or malignant changes, as they often respond to hormones as those of normally located breast [4]. Dhaoui et al. stated that about 50 cases of ectopic benign and 20 malignant breast lesions have been reported in the literature while lactating adenoma in the vulva is extremely rare [4]. Only four cases of lactating adenoma have been reported [4]. Most ectopic lactating adenomas have been noted within the age of 23 to 39 years, with measurement of 0.8 and 4 cm [6, 7]. The index patient is 26 years old and the mass measured an average of 3.5 cm.

The common origin of the ectopic breast with that of the breast at normal location can be explained by the effect of pregnancy and lactation on the ectopic breast tissue as noted in the index patient whose swelling grew significantly through pregnancy and the period of lactation. This overlapping effect of pregnancy and lactation on ectopic breast has been described by some authors [4, 6].

 An alternative theory suggests that this tissue represents the normal constituents of the anogenital area that probably do not represent true breast tissue [8, 9]. These glands are now referred to as anogenital mammary-like glands (AGMLG) by some authors [10]. Research work on the largest series of these lesions done by Kazakov et al. [10] was published in 2010. It included among others fibroadenoma of the vulva with lactational changes in a 30-year-old female [10]. 

The cytologic features of lactational lesions of ectopic breast pose special problems in diagnosis by fine needle aspiration [6]. Vulva lactating adenoma may be misdiagnosed as adenocarcinoma in frozen section and aspiration cytology if breast tissue is not anticipated [6]. 

To the best of our knowledge, this is the first case report of vulva fibroadenoma associated with lactating adenoma in a young African woman.

Complete excision of ectopic breast tissue in the vulva should be considered since the lesion can undergo malignant transformation [11, 12].

## 4. Conclusion

The rarity of vulva ectopic breast tissue can present a diagnostic challenge for both the clinician and the anatomical pathologist. It is our opinion that this case report will raise the awareness of vulva ectopic breast lesion among clinicians. Once excisional biopsy is done and the lesion confirmed histologically, the anxious patient can be reassured.
